# Whole-genome mate-pair sequencing of apparently balanced chromosome rearrangements reveals complex structural variations: two case studies

**DOI:** 10.1186/s13039-020-00487-1

**Published:** 2020-05-06

**Authors:** Ya-Qi Tan, Yue-Qiu Tan, De-Hua Cheng

**Affiliations:** 1 College of Life Science, Hunan Normal University, Changsha, 410081 China; 2 Institute of Reproduction and Stem Cell Engineering, School of Basic Medical Science, Central South University, Changsha, Hunan 410078 People’s Republic of China

**Keywords:** Apparently balanced chromosome rearrangement, Complex chromosomal rearrangement, Cryptic breakpoint, Reproductive risk, Whole-genome mate pair sequencing

## Abstract

**Background:**

Apparently balanced chromosome rearrangements (ABCRs) in non-affected individuals are well-known to possess high reproductive risks such as infertility, abnormal offspring, and pregnancy loss. However, caution should be exercised in genetic counseling and reproductive intervention because cryptic unbalanced defects and genome structural variations beyond the resolution of routine cytogenetics may not be detected.

**Case presentation:**

Here, we studied two familial cases of ABCRs were recruited in this study. In family 1, the couple suffered two abortions pregnancies and underwent labor induction. Single nucleotide polymorphism (SNP) array analysis of the aborted sample from the second pregnancy revealed a 10.8 Mb heterozygous deletion at 10q26.13q26.3 and a 5.5 Mb duplication at 19q13.41-q13.43. The non-affected father was identified as a carrier of three-way complex chromosomal rearrangement [t (6;10;19)(p22;q26;q13)] by karyotyping. Whole-genome mate-pair sequencing revealed a cryptic breakpoint on the derivative chromosome 19 (der19), indicating that the karyotype was a more complex structural rearrangement comprising four breakpoints. Three genes, *FAM24B, CACNG8,* and *KIAA0556,* were disrupted without causing any abnormal phenotype in the carrier. In family 2, the couple suffered from a spontaneous miscarriage. This family had an affected child with multiple congenital deformities and an unbalanced karyotype, 46,XY,der (11) t (6;11)(q13;p11.2). The female partner was identified as a balanced translocation carrier with the karyotype 46,XX,t (6;11)(q13;p11.2) dn. Further SNP array and fluorescent in situ hybridization (FISH) indicated a cryptic insertion between chromosome 6 and chromosome 11. Finally, whole-genome mate-pair sequencing revealed an extremely complex genomic structural variation, including a cryptic deletion and 12 breakpoints on chromosome 11, and 1 breakpoint on chromosome 6 .

**Conclusions:**

Our study investigated two rare cases of ABCRs and demonstrated the efficacy of whole-genome mate-pair sequencing in analyzing the genome complex structural variation. In case of ABCRs detected by conventional cytogenetic techniques, whole genome sequencing (WGS) based approaches should be considered for accurate diagnosis, effective genetic counseling, and correct reproductive intervention to avoid recurrence risks.

## Background

Apparently balanced chromosome rearrangements (ABCRs), including translocation, inversion, and insertion, involve exchange of genomic regions between non-homologous chromosomes without the gain or loss of genetic material. Simple ABCR is a two-breakage rearrangement, whereas complex chromosomal rearrangement (CCR) involves chromosomal abnormalities with three or more breakpoints. CCRs are rare in the population, with only around 380 cases reported till date [1, 2]. Although most ABCRs are associated with a normal phenotype, they show high reproductive risks such as infertility, recurrent spontaneous miscarriage, and offspring with developmental defects and so on [3–5].

Reproductive risk increases with increase in the number of breakpoints, and the particular rearrangements associated with peculiar mis-segregation mode in meiosis. For example, in reciprocal translocation, small translocated segments are prone to adjacent-1 segregation, small centric segments are apt for adjacent-2 segregation, and a small quadrivalent is usually associated with 3:1 disjunction. Indeed, some translocation carriers have more than 50% risk of bearing an abnormal child, whereas others have less than 1% risk. Therefore, it is crucial to accurately diagnose the karyotype of ABCR carriers with normal phenotype to determine the reproductive risk and choose an appropriate approach to avoid birth defects, such approaches include spontaneous pregnancy combined with prenatal diagnosis, preimplantation genetic testing, and use of donor sperm or egg.

Accurate cytogenetic diagnosis depends on the resolution of the testing techniques used. Previously, identification of ABCRs and further breakpoint confirmation were mainly based on high-quality G-banding of the metaphase chromosomes. However, due to the limited resolution of conventional cytogenetic technologies, only large structural rearrangements (> 5 Mb) are identified. As a result, submicroscopic structural rearrangements could remain undetected, even when high-resolution banding technology is used. Molecular testing techniques such as FISH, CMA, and WGS have greatly improved the ability to identify ABCRs [6–10]. Recent studies have reported that WGS can detect extremely complex balance rearrangements, including genome structural variations at the molecular level, which otherwise remain detected by conventional G banding techniques [11].

In this study, we analyzed the structural features of two cases of CCR using low pass whole-genome mate-pair sequencing to perform risk evaluation of the ABCR carriers and to examine complex genome structure variations in the ABCRs detected by conventional cytogenetic technology.

## Case presentation

**Fig. 1:**
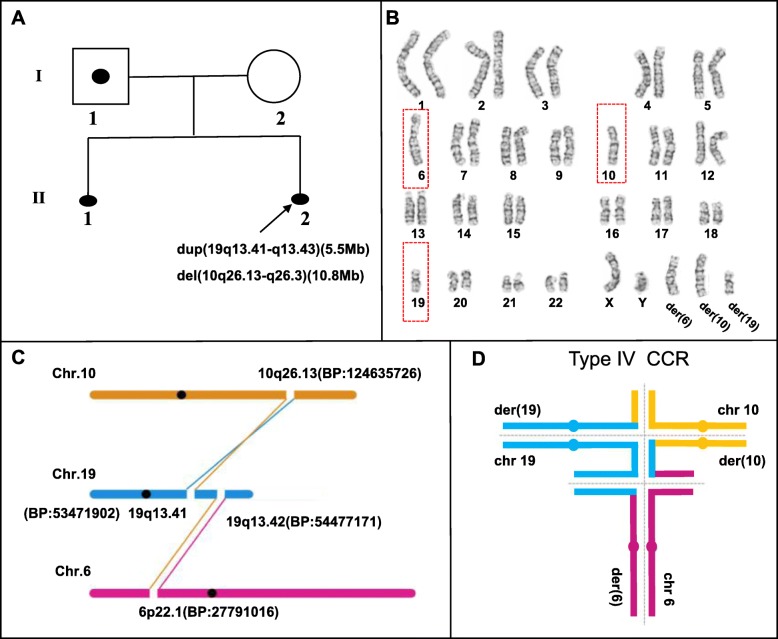
Molecular cytogenetic analysis for Family 1. **a** The pedigree of family 1 with the proband (II-2) indicated by an arrow. Black spot, induced labor; open symbols, unaffected individuals; square with black spots, male carriers; circles, females. **b** The husband in family 1 was a carrier for a complex translocation between chromosome 6, 10, and 19 by G-banding analysis. **c** Whole genome DNA sequencing of peripheral blood from the carrier revealed four breakpoint rearrangement karyotypes. The genetic material from chromosomes 6, 10, and 19 is indicated as purple, yellow, and blue lines, respectively. BP stands for breakpoint. **d** Type IV CCR Hexavalent Configurations. type IV CCR, as refined by whole-genome mate-pair sequencing in the current study. The additional breakpoint as well as possible recombination at the “middle segment” in type IV CCR increases the percentage of unbalanced gametes, and subsequent reproductive risk. Genetic material from chromosomes 6, 10, and 19 is shown as purple, yellow, and blue lines, respectively

Family 1: A couple (I-1 and I-2 in Fig. 1a), with the husband aged 32 years and the wife aged 27 years, visited our hospital for genetic counseling and birth guidance. They had conceived two natural pregnancies, which were terminated due to the observation of abnormal fetus during routine prenatal check-ups. In the first pregnancy, B-ultrasonography at week 13 of pregnancy revealed fetal growth retardation, as the fetus size was equivalent to that at 12 W, NT thickening and a small amount of regurgitation in the reverse tricuspid valve of the venous catheter were also detected. This pregnancy was terminated at 13 weeks. In the second pregnancy, four-dimensional B-ultrasonography at week 20 of pregnancy revealed a thickened fetal neck skin fold, lymphatic hydrocystoma of the neck, wide eye distance, short nasal bone, and hydramnios. Amniocentesis and chromosome microarray analysis of amniotic fluid cells (AFC) were performed. The pregnancy was terminated after the prenatal diagnosis showed an abnormal result.

**Fig. 2:**
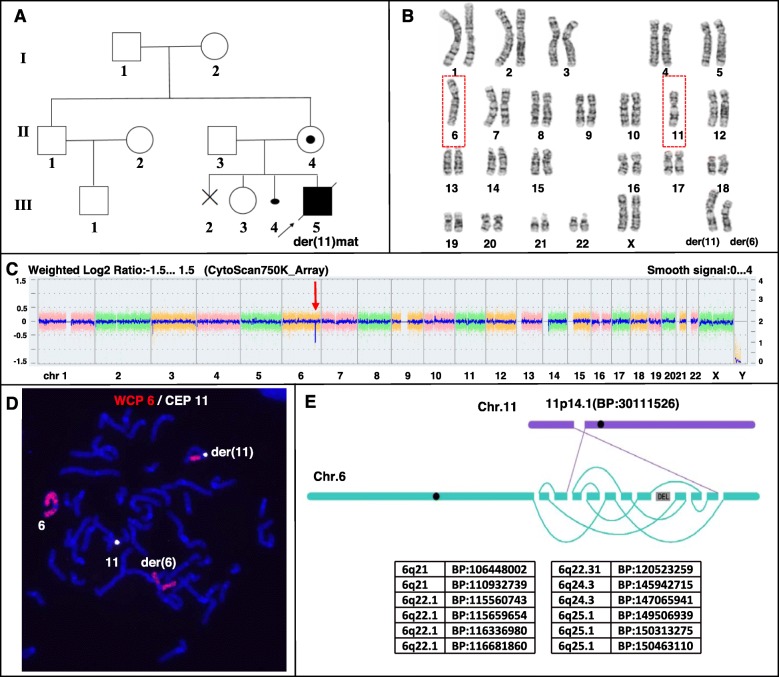
Molecular cytogenetic analysis for Family 2. **a** The pedigree of family 2. **b** The wife in family 2 showed recombination chromosome 6 and 11 by G-banding analysis. **c** The results of the CytoScan750K_Array. The figure shows the signal of loss (Red arrow) in chromosome 6. **d** The results of WCP 6 (Red signal)/CEP 11 (White signal) FISH show insertion translocation between chromosome 6 and 11. (E) Whole genome DNA sequencing of peripheral blood from the carrier revealed thirteen breakpoints and rearrangement in the karyotypes with a cryptic deletion on chromosome 6. Genetic material from chromosomes 6 and 11 are indicated as purple and blue lines, respectively. BP stands for breakpoint

Family 2: This couple (II-3 and II-4 in Fig. 2a), with both partners aged 36 years, visited our hospital to conceive a healthy baby. They had four natural pregnancies after marriage. The first and the third pregnancies were naturally miscarried in the first trimester due to unknown reasons. The second pregnancy concluded with the delivery of a healthy daughter having a normal chromosome karyotype. During the fourth pregnancy, the couple did not carry out routine ultrasonography examination and invasive prenatal testing; this pregnancy led to the birth of a boy with anal atresia, congenital heart disease, and penile scrotal transposition, who survived only for over 100 days after birth.

## Materials and methods

Karyotyping analysis was performed for eight individuals including I-1 and I-2 in family 1 and I-1, I-2, II-3, II-4, III-3, and III-5 in family 2. SNP array analysis of II-2 in family 1 was performed. II-4 in family 2 underwent SNP array analysis and FISH testing. WGS was performed for both I-1 in family 1 and II-4 in family 2.

Karyotyping analysis, FISH, and SNP array were performed as described in our previous study [12]. Briefly, metaphase G-banding at the 400–550 band level was performed using peripheral blood lymphocytes. Metaphase FISH was performed using whole chromosome painting probe 6 (WCP6) and centromere-enumeration probe 11 (CEP 11) (Abbott-Vysis, Des Plaines, IL, USA). Single nucleotide polymorphism (SNP) microarray analysis was performed using Cytoscan 750 K chip (Affymetrix, Santa Clara, CA, USA) as previously reported [13]. The data were analyzed using chromosome analysis software ChAS (Affymetrix, Santa Clara, CA, USA).

DNA was isolated from peripheral blood lymphocytes using a QIAamp® DNA blood midi kit (QIAGEN, Hilden, Germany). Low-coverage whole genome massively parallel sequencing was performed in two carriers for excluding the cryptic chromosome structural rearrangements. Briefly, a non-size selected mate-pair library was prepared using ~ 3 μg of genomic DNA and then subjected to 50-bp-end multiplex sequencing on the Illumina HiSeq™ X10 platform. After automatically removing adaptor sequences and low-quality reads, high quality paired-end reads were aligned to the NCBI human reference genome (GRCh37/hg19) using SOAP2. Uniquely mapped reads were selected for subsequent analysis as previously described [12]. After bioinformatics analysis, we obtained the breakpoint regions were identified through bioinformatic analysis.

## Results

Family 1: Chromosome microarray (CMA) testing of amniotic fluid cells (AFC) using SNP 750 K revealed that the fetus had an unbalanced chromosome rearrangement between the chromosomes 10 and 19, i.e. arr (hg19)10q26.13q26.3(124,625,736-135,426,386) × 1 (10.8 Mb deletion) and arr (hg19)19q13.41-q13.43(53,487,026-58,956,816) × 3 (5.5 Mb duplication), respectively. G-banding karyotype analysis showed that the female partner had a normal karyotype, whereas the male partner with the karyotype 46,XY,t (6;10;19)(p22;q26;q13) had a complex translocation between the chromosomes 6, 10, and 19 (Fig. 1b). To precisely determine the karyotype, further WGS analysis was performed and two rearrangement breakpoints on chromosome 19 were detected:, 19q13.41 and 19q13.42, respectively (Fig. 1c). The intermediate segment 19q13.41-19q13.42 of the translocated chromosome 19 was inserted into the derivative chromosome 10. Finally, the four breakpoints of the chromosomes 6, 10 and 19 were confirmed to be involved in the complex balanced rearrangement of karyotypes, and with disruption in three genes, *FAM24B, CACNG8*, and *KIAA0556*.

Family 2: G-banding karyotype analysis of III-5 at his birth detected a maternal unbalanced translocation. The male partner (II-3) had a normal karyotype, whereas the female partner (II-4) with 46,XX,t (6;11)(q13;p11.2) dn karyotype showed a de novo reciprocal translocation between the chromosomes 6 and 11, and her karyotype was interpreted as 46,XX,t (6;11)(q13;p11.2) dn. (Fig. 2b). Both parents (I-1 and I-2) of the female partner both had a normal karyotype.

Fluorescence in situ hybridization (FISH) analysis of the female partner (II-4) with a chromosome 6 painting probe revealed that an intermediate segment 6q21-q25.1 was inserted into 11p14.1, the middle region of the short arm of chromosome 11 (Fig. 2d). WGS was performed to precisely map the insertion rearrangement, revealing 12 breakpoints in the 106.448–150.463 Mb region of the long arm of chromosome 6 (6q21-q25.1) that were associated with a heterozygous deletion in the 145.943–147.066 Mb region of the long arm of chromosome 6 (Fig. 2e). Chromosome microarray (CMA) using SNP (750 K, Affymetrix) showed a cryptic heterozygous microdeletion in the 146–147 Mb region of chromosome 6q in the female partner (Fig. 2c), confirming the reliability of WGS analysis. The karyotypes of the parents of the female partner and her healthy daughter were normal, whereas her affected son inherited the maternal derivative chromosome 11, thus the normal phenotype of the daughter and the abnormal phenotype of the son were attributable to the karyotype. Surprisingly, 13 breakpoints in the chromosomes 6 and 11 were identified in the karyotype of the female partner, indicating that WGS can detect complex genome structural variations. Due to insufficient DNA, precise breakpoint analysis by PCR was not carried out.

## Discussion

Detection of ABCR carriers is very important in ABCRs with normal phenotype owing to a high risk of recurrent spontaneous miscarriages and birth defects [11, 12, 14]. In this study, two couples from different families had a history of recurrent adverse pregnancies, suggesting the necessity for cytogenetic testing. Karyotype analysis and further WGS revealed that the patients harbored complex chromosomal rearrangements. Our study demonstrates the advantages of WGS in karyotype analysis.

Submicroscopic chromosomal abnormalities or cryptic rearrangements with similar chromosome band modes may remain undetected by conventional techniques due to the limitations of testing sensitivity [11]. Our study showed that low-coverage WGS technique has all the advantages of the conventional testing techniques; as it can detect the chromosomal rearrangements of large segments similar to traditional karyotype analysis, and can also detect submicroscopic copy number variations similar to a chromosome microarray. In particular, it can detect cryptic genome structure variations.

Insertion is a rare three-break rearrangement with an incidence of only 1/80,000, as reported in previous studies [15]. However, a submicroscopic insertion was found in each family included in this study. In family 1, a fragment of chromosome 19 was inserted into chromosome 10, while in family 2, a large fragment of chromosome 6 was inserted into chromosome 11. Type IV CCR configurations must have formed during the pachytene stage of meiosis for the carrier in family 1 (Fig. 1d) [1, 16]. The CCR with an inserted middle segment can produce new rearrangements and result in higher reproductive risk, increased unbalanced gamete production, and consequently, affected offspring. Our study indicates that chromosomal insertion might be much more common after the testing resolution is increased. As insertion itself can lead to the risk of spontaneous abortion and birth defects, the reproductive risk was found to be higher in this study than in the previous testing results showing a three-way translocation in family 1 and a simple translocation in family 2.

The identification of more submicroscopic chromosomal abnormalities might elucidate some unexplained spontaneous miscarriages and birth defects, and could provide a basis for accurate genetic counseling. However, improvement of testing sensitivity may also give rise to new problems in the interpretation of results and clinical genetic counseling, such as expressing the complexity of chromosomal structure rearrangement due to a lack of WGS based nomenclature, determining the pathogenicity of small fragments of CNV, and explaining alterations in the pattern of meiotic recombination due to cryptic genome structural rearrangements. Thus, additional data on genome structural variations using WGS technique are needed to answer these questions.

## Conclusions

In conclusion, we present two rare cases of ABCRs with cryptic insertion and minute imbalance structural abnormalities identified by whole-genome mate-pair sequencing. Our study showed that WGS breakpoint studies can facilitate improved understanding of complex genomic structural variations. In the future, the incidence of cryptic CCR should be investigated in a large cohort of ABCR carriers diagnosed by traditional G banding karyotype.

## Data Availability

The datasets generated and analyzed during the current study are available from the corresponding author on reasonable request.

## Abbreviations

WGS: Whole-genome sequencing
SNP: Single-nucleotide polymorphism
FISH: Fluorescence in situ hybridization
CNV: Copy number variation
PCR: Polymerase chain reaction
WCP: Whole chromosome painting
